# CD4^+^ T cells stratification unveils a dynamic pathogen landscape in severe pneumonia: a targeted next-generation sequencing-based cohort study

**DOI:** 10.3389/fcimb.2025.1742311

**Published:** 2025-12-18

**Authors:** Shuaishuai Wang, Yue Yu, Che Lihe, Luyao Sun, Na Du, Zhang Minglei

**Affiliations:** 1 Department of Xinmin Orthopedic, China-Japan Union Hospital of Jilin University, Changchun, China; 2 Department of Infectious Diseases, Infectious Diseases and Pathogen Biology Center, The First Hospital of Jilin University, Changchun, China

**Keywords:** BALF, CD4, infection, severe pneumonia, TNGS

## Abstract

**Background:**

The host’s immune status is a critical determinant of the pathogen profile in pulmonary infections, but quantitative indicators to delineate this dynamic association are lacking. This study aimed to use CD4^+^ T cells count as an objective parameter to systematically characterize the evolution of the pathogen profile in bronchoalveolar lavage fluid-targeted next-generation sequencing (BALF-tNGS) from patients with severe pneumonia.

**Methods:**

We retrospectively analyzed 286 adult patients who underwent BALF-tNGS for severe pneumonia. Patients were stratified into four strata based on CD4^+^ T cells count: severe immunosuppression, moderate immunosuppression, low-normal immunity, and immunocompetent. Cochran-Armitage trend test, cluster analysis, and Kruskal-Wallis test were used to analyze the associations between CD4^+^ T cells strata and pathogen detection rates/co-infection complexity.

**Results:**

The detection rates of *Pneumocystis jirovecii* (*PJP*) and Cytomegalovirus (CMV) showed a highly significant increasing trend with decreasing CD4^+^ T cells strata (*p* < 0.05). CD4^+^ T cells count < 100 cells/μL was the most significant risk factor for PJP infection (detection rate 39.6%). Meanwhile, as the CD4^+^ T cells count decreased, the number of pathogen species detected in the patient’s BALF significantly increased, with a statistically significant difference between groups (*p* < 0.05). The median number in the severe immunosuppression stratum was significantly higher than that in the immunocompetent stratum.

**Conclusion:**

The CD4^+^ T cells count quantitatively defines the pathogen ecology in pulmonary infections. A CD4^+^ T cells count < 100 cells/μL represents a critical threshold for opportunistic infections and complex co-infections. These findings support the integration of CD4^+^ T cells counting into the initial assessment framework for severe pneumonia to guide precise empirical therapy and diagnostic strategies.

## 1 Introduction

Severe pneumonia is a life-threatening condition with high global morbidity and mortality, posing a significant challenge to clinicians. The cornerstone of effective management lies in the timely and accurate identification of the causative pathogens (Kyu et al., 2022; Qu et al., 2022). However, conventional diagnostic methods—including culture and pathogen-specific polymerase chain reaction(PCR)—often have limitations in terms of sensitivity and turnaround time, making it difficult to provide a comprehensive pathogen profile, particularly in complex cases (Gaston et al., 2022; Zhang et al., 2024).

The host’s immune status is a critical determinant of the pathogen profile in pulmonary infections (Crossen et al., 2023; Husmann et al., 2025). Traditionally, patients have been simply dichotomized as immunocompetent or immunodeficient based on clinical history, such as HIV infection, solid organ transplantation, or active malignancy (SeyedAlinaghi et al., 2022; Xue et al., 2023). Although this approach is easy to implement, it oversimplifies the situation and fails to reflect the degree of immune dysfunction. It lacks a quantitative basis for stratifying infection risk and guiding preemptive prophylactic therapy, particularly in patients with subtle or undefined immunodeficiencies. CD4^+^ T cells, as central regulators of adaptive immunity, serve as a key quantitative biomarker for assessing a host’s cellular immune function (Sun et al., 2023). Its utility in risk stratification has been well established (Zlei et al., 2022). However, in the broader population of patients with severe pneumonia, including those with non-HIV-related immunosuppression, the dynamic relationship between CD4^+^ T cells count and the wider pathogen ecology has not yet been systematically investigated or elucidated.

The advent of BALF-tNGS has refined pathogen detection strategies by enabling the targeted enrichment and high-throughput sequencing of predefined pathogen-associated nucleic acids in clinical samples (Hong et al., 2023; Murphy et al., 2023; Yin et al., 2024). This targeted approach allows for the efficient and sensitive detection of pathogens within a predetermined panel (including atypical and fastidious organisms), as well as the identification of co-infections, thereby providing an efficient solution for the rapid and precise delineation of the pathogen profile in specific clinical contexts (Lin et al., 2023; Sun et al., 2024; Colman et al., 2025). Therefore, this study aimed to leverage the precision of CD4^+^ T cells count and the comprehensive nature of BALF-tNGS to delineate a quantitative and dynamic landscape of the pathogen profile in the BALF of patients with severe pneumonia. We hypothesized that a descending gradient of CD4^+^ T cells count would be associated with distinct and progressively complex shifts in pathogen prevalence, thereby revealing critical risk thresholds for specific opportunistic infections. By moving beyond a simple binary classification, we sought to establish CD4^+^ T cells count as a key variable for optimizing initial empirical antimicrobial therapy and refining diagnostic pathways in severe pneumonia.

## 2 Methods

### 2.1 Study design and population

This study is a retrospective single-center observational cohort study. We consecutively screened adult patients (age ≥ 18 years) who were admitted for severe pneumonia and underwent BALF-tNGS between January 1, 2024, and December 31, 2024.

Inclusion criteria for this study were: (1) clinical diagnosis of severe pneumonia; (2) successful bronchoscopy with acquisition of qualified BALF samples; (3) availability of complete peripheral blood lymphocyte subset test results (including CD4^+^ T cells count). Exclusion criteria were: (1) missing CD4^+^ T cells count data; (2) incomplete clinical medical records preventing the acquisition of key baseline characteristics or outcome data.

The study protocol was approved by the Institutional Ethics Review Board of our hospital, and the requirement for informed consent was waived by the ethics committee because this retrospective study analyzed anonymized clinical data without any additional intervention.

### 2.2 Data collection

Two trained researchers independently extracted data from the electronic medical record system and performed cross-verification to ensure accuracy. The collected data included:

Demographics: age and sex.

Clinical symptoms and signs: fatigue, headache, shortness of breath, etc., upon admission.

Comorbidities and immune status: hypertension, diabetes, chronic kidney disease, malignancy, autoimmune diseases, hematological diseases, etc.

Laboratory parameters: The most recent peripheral blood test results prior to BALF-tNGS submission were collected, including: white blood cell count, neutrophil percentage, lymphocyte percentage, C-reactive protein, procalcitonin, interleukin-6, and key lymphocyte subset absolute count.

Etiological test results: The BALF-tNGS results served as the primary basis for pathogen identification. Results from conventional microbiological tests, such as blood culture, sputum culture, fungal (1,3)-β-D-glucan test (G test), and galactomannan test (GM test), were also recorded to aid clinical interpretation.

Treatment and outcomes: Initial and subsequent antimicrobial therapy, length of hospital stay, and discharge status.

### 2.3 Definition of CD4^+^ T cells immunological

Strata based on established immunological stratification criteria in infectious diseases, patients were categorized into four ordinal immunological strata according to their baseline CD4^+^ T cells count:

Stratum 1 (Severe immunosuppression): CD4^+^ T cells < 100 cells/μL

Stratum 2 (Moderate immunosuppression): 100 ≤ CD4^+^ T cells < 200 cells/μL

Stratum 3 (Low-normal immunity): 200 ≤ CD4^+^ T cells < 500 cells/μL

Stratum 4 (Normal immunity): CD4^+^ T cells ≥ 500 cells/μL

### 2.4 Pathogen data analysis

All pathogens detected (including bacteria, viruses, fungi, and atypical pathogens) were extracted from the BALF-tNGS reports. During data analysis, the sequenced reads are aligned against the authoritative NCBI RefSeq database for pathogen identification. For result interpretation, specific positive thresholds are applied, provided the corresponding pathogen is not detected in the negative control. Specifically, the required number of unique reads is >50 for bacteria, >3 for fungi, >10 for viruses, and >100 for parasites. Establishing these thresholds effectively distinguishes true pathogen infection from background noise, thereby ensuring the specificity of the results and the reliability of clinical guidance. Pathogens were categorized to facilitate the analysis. The analysis focused on opportunistic pathogens associated with immune status.

### 2.5 Statistical analysis

All statistical analyses were performed using the *R* language(version 4.5.1), with the significance level set at a two-sided α = 0.05. Descriptive Statistics: Continuous variables conforming to a normal distribution are presented as mean ± standard deviation and were compared between groups using analysis of variance. Non-normally distributed continuous variables are presented as median (interquartile range) and were compared using the Kruskal-Wallis H test. Categorical variables are presented as frequencies and percentages and were compared using the chi-square test or Fisher’s exact test, as appropriate. Trend Analysis: The Cochran-Armitage trend test was employed to analyze the changes in the detection rates of specific pathogens across the CD4^+^ T cells strata, assessing for the presence of a significant linear trend. Analysis of Co-infection Complexity: The number of pathogen species detected via BALF-tNGS for each patient was calculated as an indicator of co-infection complexity. Differences in the number of pathogen species among the CD4^+^ T cells strata were compared using the Kruskal-Wallis H test.

### 2.6 Study population and baseline characteristics

**Figure 1:**
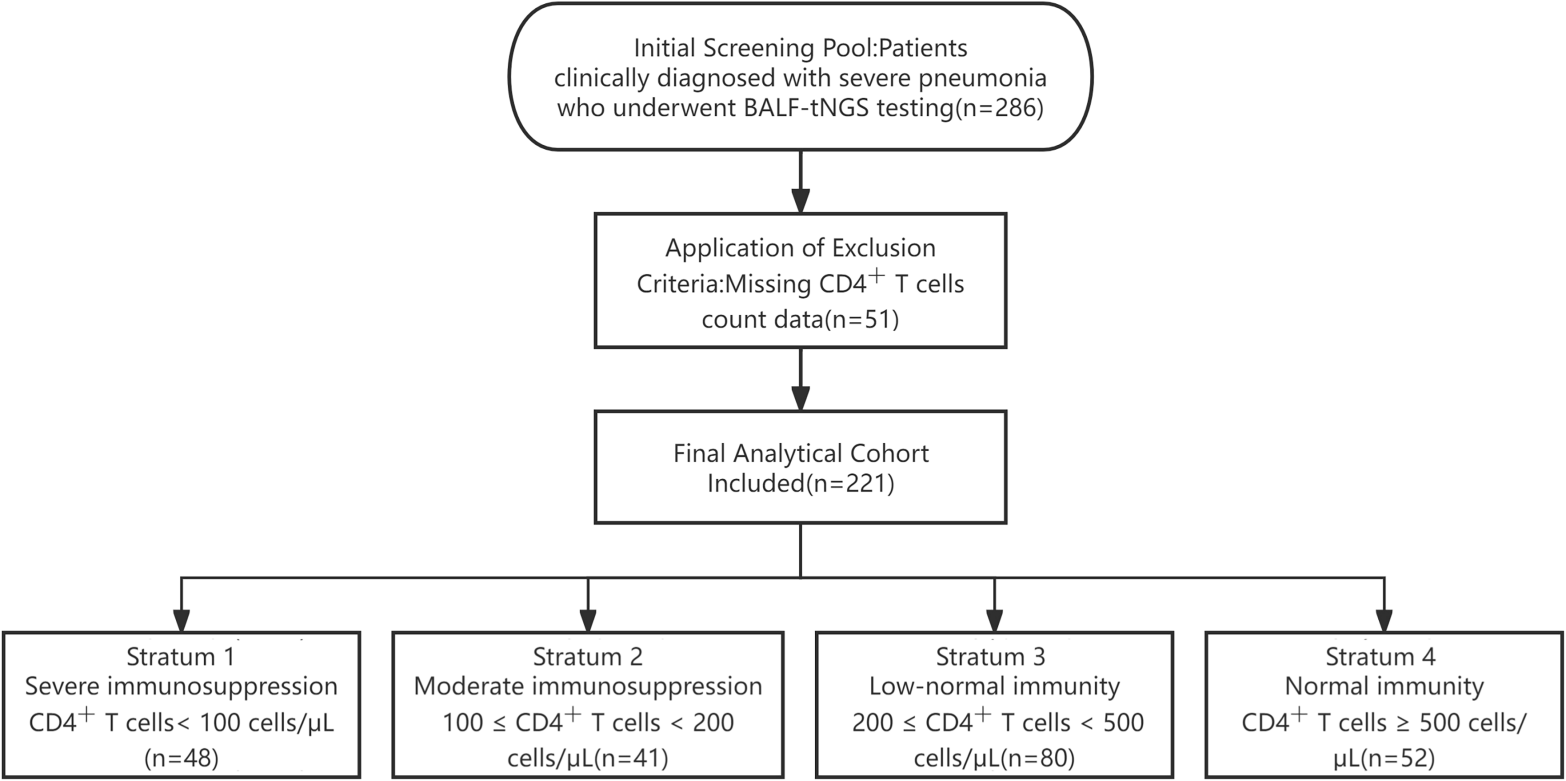
Flowchart of patient screening, enrollment, and stratification based on CD4^+^ T cells count. BALF, bronchoalveolar lavage fluid; tNGS, targeted next-generation sequencing.

According to the inclusion and exclusion criteria, a total of 286 patients clinically diagnosed with severe pneumonia and undergoing BALF-tNGS were initially screened. As shown in **Figure 1**, after applying the exclusion criteria (missing CD4^+^ T cells count data, *n* = 51; incomplete key clinical data, *n* = 14), 221 patients were included in the final analytical cohort. The cohort was stratified into four distinct groups based on baseline CD4^+^ T cells count: Stratum 1 (Severe Immunosuppression, CD4^+^ T cells < 100 cells/μL, *n* = 48), Stratum 2 (Moderate Immunosuppression, 100 ≤ CD4^+^ T cells < 200 cells/μL, *n* = 41), Stratum 3 (Low-Normal Immunity, 200 ≤ CD4^+^ T cells < 500 cells/μL, *n* = 80), and Stratum 4 (Immunocompetent, CD4^+^ T cells ≥ 500 cells/μL, *n* = 52).

No significant differences were observed in age, gender, or the distribution of major underlying comorbidities among the four groups, indicating good comparability. However, a statistically significant difference in IL-6 levels was found across the groups (*p* = 0.008), with the Normal immunity stratum (Stratum 4) exhibiting the highest IL-6 concentration (**Table 1**). This paradoxical finding suggests a complex interplay between cellular immunity and the inflammatory response, where immunocompetent patients mount a more robust inflammatory reaction to infection. In contrast, immunosuppressed patients may exhibit blunted inflammatory responses despite having a severe infection.

**Table 1:** Baseline characteristics of the study population stratified by CD4^+^ T cells count.

Characteristic	Stratum1 (CD4^+^ T cells < 100, *n* = 48)	Stratum2 (100 ≤CD4^+^ T cells < 200, *n* = 41)	Stratum3 (200 ≤CD4^+^ T cells < 500, *n* = 80)	Stratum4 (CD4^+^ T cells ≥ 500, *n* = 52)	*P-value*
Age (years), median (IQR)	64.5 (52.5-76.0)	67.0 (58.5-76.0)	62.0 (49.5-73.5)	59.0 (44.5-71.5)	0.685
Male, *n* (%)	32 (66.7)	29 (70.7)	56 (70.0)	36 (69.2)	0.499
Comorbidities, *n* (%)					
Hypertension	20 (41.7)	17 (41.4)	32 (40.0)	16 (30.8)	0.432
Diabetes	12 (25.0)	9 (21.9)	20 (25.0)	8 (15.4)	0.714
Malignancy	7 (14.6)	13 (31.7)	16 (20.0)	4 (7.7)	0.351
Inflammatory Markers, median (IQR)					
CRP (mg/L)	89.5 (25.3-172.3)	104.3 (61.5-217.5)	63.3 (25.0-98.6)	66.2 (21.1-95.5)	0.078
PCT (ng/mL)	2.27 (0.08-3.53)	1.78 (0.10-3.59)	3.17 (0.05-2.60)	2.15 (0.05-3.41)	0.562
IL-6 (pg/mL)	47.2 (13.0-169.9)	57.4 (22.4-128.5)	77.3 (19.2-165.1)	82.8 (42.6-222.5)	0.008

### 2.7 Distribution trends of key pathogens across CD4^+^ T cells strata

**Figure 2:**
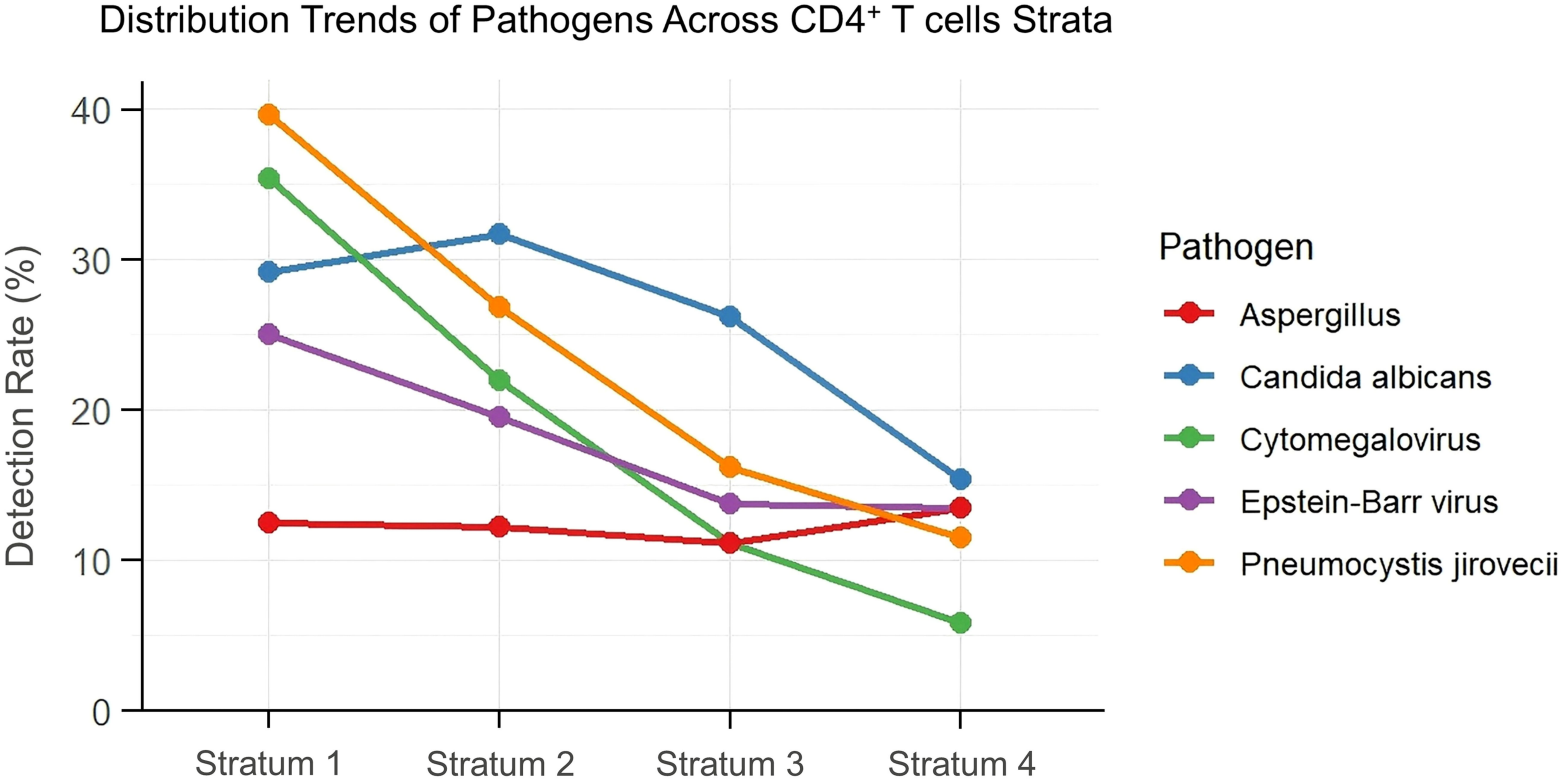
Distribution trends of opportunistic pathogens across CD4^+^ T cells strata based on BALF-tNGS detection. (Cochran-Armitage trend test, *PJP*, *p* < 0.05; CMV, *p* < 0.05; *Candida, p* = 0.067; *Aspergillus, p* = 0.931; EBV, *p* = 0.085).

BALF-tNGS detected a variety of pathogens. Trend analysis was performed for several important opportunistic pathogens. As shown in **Figure 2**, the detection rates of *Pneumocystis jirovecii* (*PJP*) and Cytomegalovirus (CMV) demonstrated a significant increasing trend with decreasing CD4^+^ T cells strata(Cochran-Armitage trend test, *PJP*, *p* < 0.05; CMV, *p* < 0.05). In Stratum 1 (CD4^+^ T cells < 100 cells/μL), the detection rate of *PJP* was as high as 39.6%, while that of CMV was 35.4%. In Stratum 4 (CD4^+^ T cells ≥ 500 cells/μL), the detection rates of *PJP* and CMV decreased to 11.5% and 5.8%, respectively. In contrast, no significant trend variations in detection rates were observed for Epstein-Barr virus (EBV), *Aspergillus*, or *Candida* across the different CD4^+^ T cells strata.

### 2.8 Overall pathogen profile stratified by CD4^+^ T cells status

**Figure 3:**
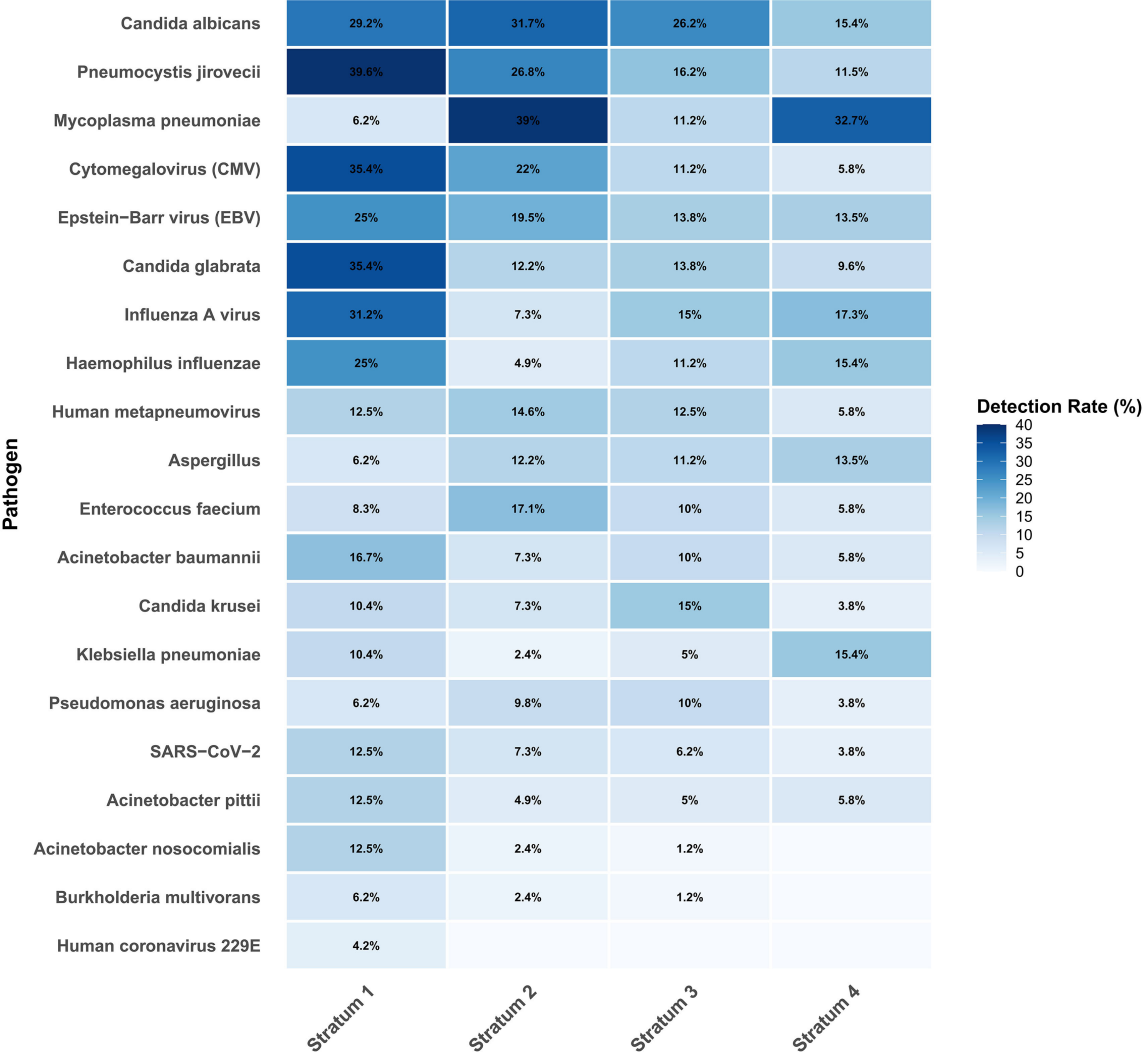
Pathogen detection frequency stratified by CD4^+^ T cells count.

To provide a global view of pathogen distribution, a heatmap was generated (**Figure 3**). The heatmap clearly demonstrates that opportunistic pathogens, represented by *PJP* and CMV, formed distinct enrichment clusters predominantly in Stratum 1 and Stratum 2 (left side). In contrast, common bacteria such as *Pseudomonas aeruginosa* and *Klebsiella pneumoniae* were distributed relatively evenly across all strata. Furthermore, the visualization intuitively suggests a more complex pathogen profile, indicative of greater co-infection complexity, among patients in the lower CD4^+^ T cells strata.

### 2.9 Analysis of co-infection complexity

**Figure 4:**
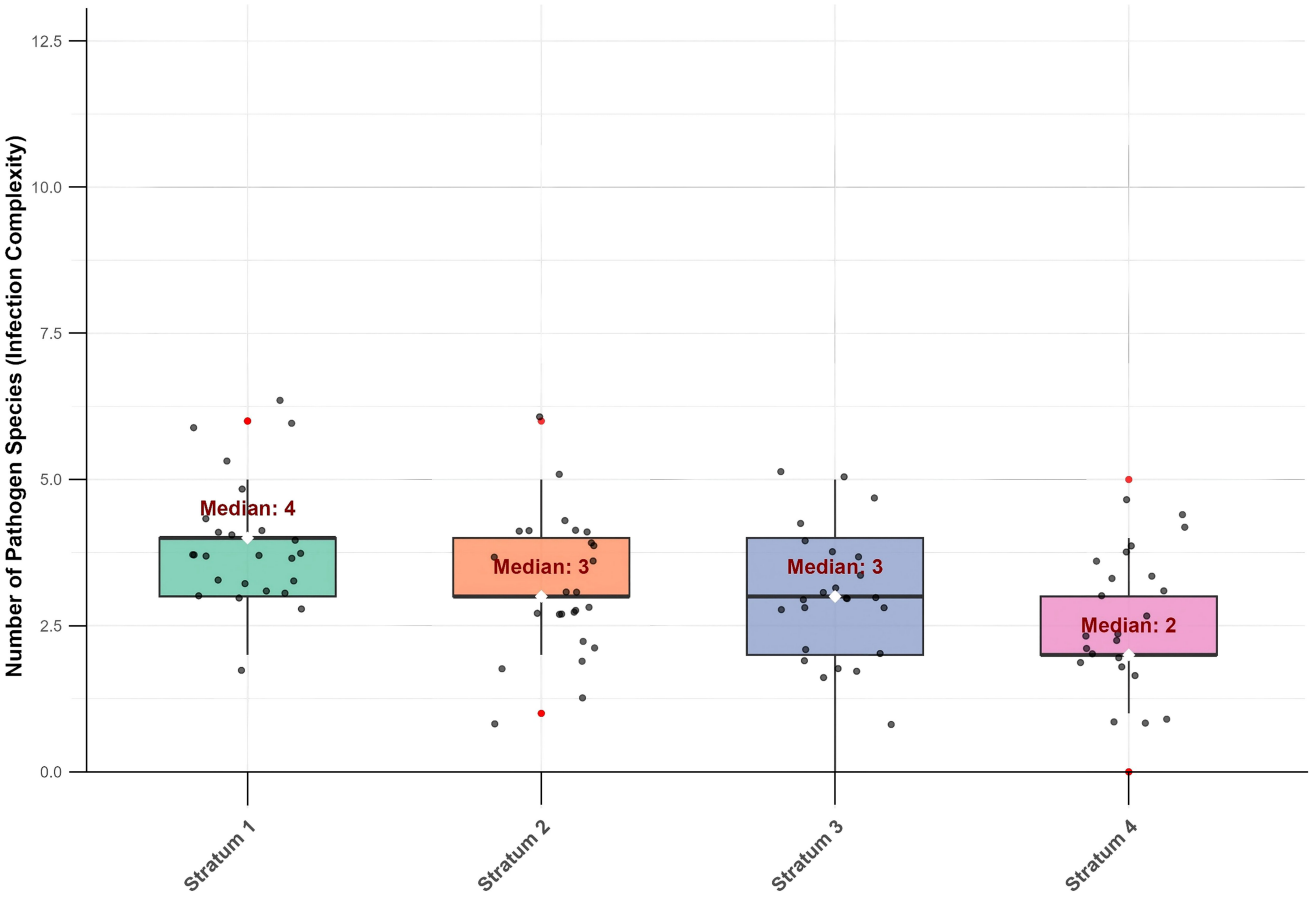
Co-infection complexity across CD4^+^ T cells immunological strata. (Kruskal-Wallis test, *p* < 0.05).

We further quantified the co-infection complexity for each patient, defined as the number of distinct pathogen species detected. The analysis revealed a significant increase in co-infection complexity with the severity of immunosuppression (*p* < 0.05). The median number of pathogen species was 4 in Stratum 1, which was significantly higher than the median of 2 species observed in Stratum 4 (**Figure 4**).

### 2.10 Clinical outcomes

**Figure 5:**
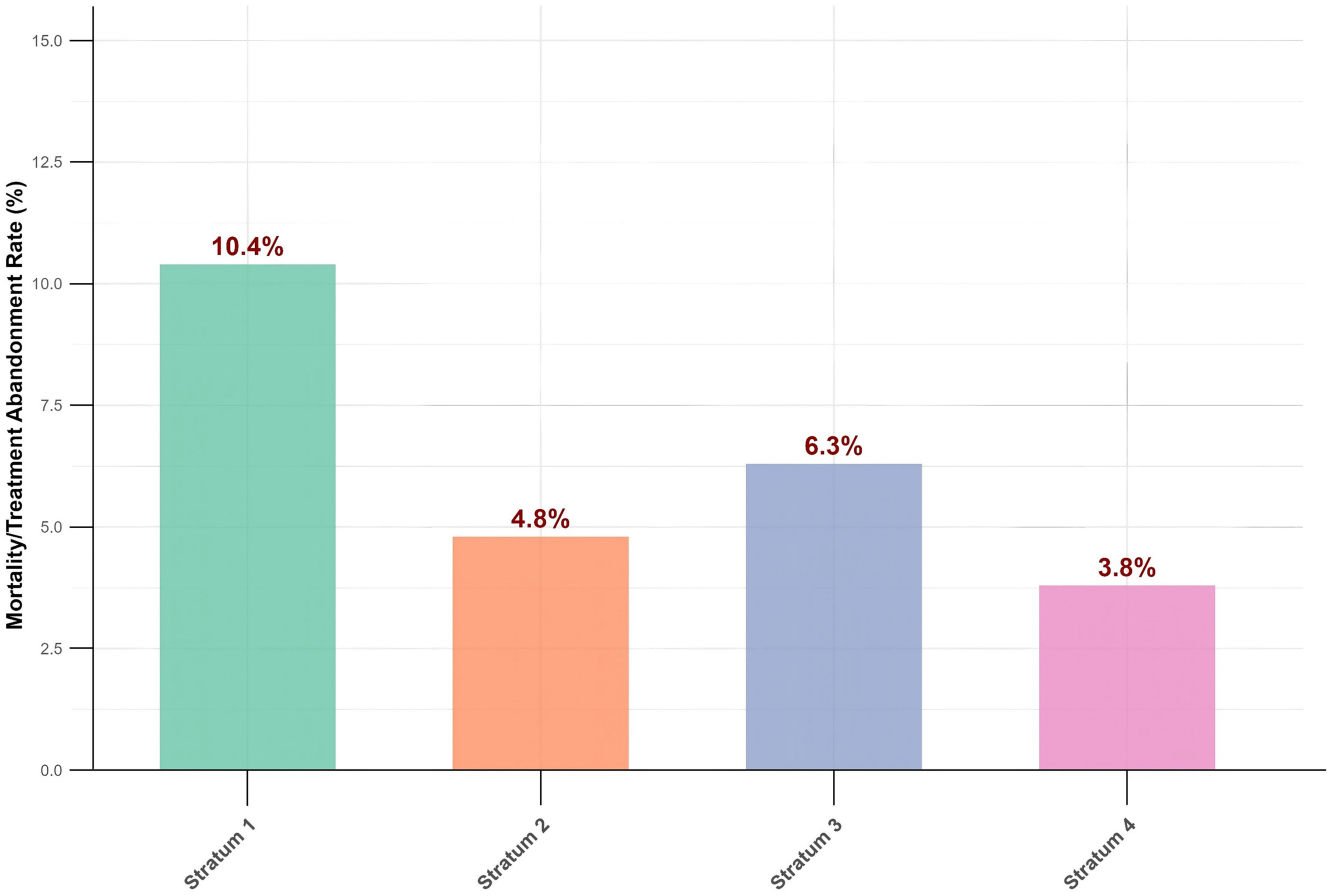
Comparison of mortality and treatment abandonment rates across CD4^+^ T cells strata. (Chi-square test, *p* = 0.166; Fisher’s exact test, *p* = 0.198).

Regarding clinical outcomes, the mortality/treatment withdrawal rate in Stratum 1 (severe immunosuppression) was 10.4%, which was higher than that in the other strata. The observed rates were 4.8% in Stratum 2, 6.3% in Stratum 3, and 3.8% in Stratum 4. Although Stratum 1 showed the highest rate, the differences among groups were not statistically significant (Chi-square test, *p* = 0.166; Fisher’s exact test, *p* = 0.198) (**Figure 5**).

## 3 Discussion and conclusion

By stratifying patients with severe pneumonia based on the objective immunological parameter of CD4^+^ T cells count and leveraging BALF-tNGS, this study provides the evolution of the pulmonary pathogen profile along a gradient of declining host cellular immune function. Our principal findings can be summarized in three key points. First, the risk of infection with specific opportunistic pathogens, particularly *PJP* and CMV, demonstrates a significant inverse correlation with CD4^+^ T cells count, exhibiting a clear threshold effect. Second, the probability of complex polymicrobial infections increases with the severity of immunosuppression. Third, the intensity of the inflammatory response, as represented by IL-6, also varies according to the immune status defined by the CD4^+^ T cells count. These findings have important implications for the precise diagnosis and treatment of severe pneumonia.

### 3.1 CD4^+^ T cells count as a quantitative predictor of opportunistic infection risk

This study found that the detection rates of *PJP* and CMV increased sharply in the group with CD4^+^ T cells count < 100 cells/μL, a finding with significant clinical relevance. While this phenomenon is well-established in people living with HIV (Tang et al., 2025), our findings demonstrate that the same strong association exists in a broader cohort of patients with severe pneumonia, irrespective of HIV status. This association is equally applicable to non-HIV immunosuppressed patients, such as those with malignancies or autoimmune diseases. A CD4^+^ T cells count < 100 cells/μL can be regarded as a critical “risk threshold.” When a patient falls below this level, clinicians should maintain a high index of suspicion for *PJP* and CMV infection, even in the absence of a typical HIV history. This finding advances the traditional qualitative concept of immunodeficiency into a new phase of risk quantification centered on CD4^+^ T cells, providing precise guidance for empirical antimicrobial therapy. In contrast, pathogens such as EBV and *Aspergillus* did not demonstrate a significant trend. This may be related to their complex characteristics as colonizing organisms or reactivated latent infections, whose clinical significance requires comprehensive interpretation in conjunction with host factors and other diagnostic findings.

### 3.2 Microbiological manifestation of immune exhaustion

Another key finding of this study is the significant increase in the number of pathogen species detected in BALF as the CD4^+^ T cell stratum decreases. This provides direct visual evidence, from a microbiological perspective, of the progressive loss of immune control in the host. In immunocompetent individuals, the immune system can effectively suppress most opportunistic pathogens, and infections are typically dominated by a single or a limited number of pathogens. In contrast, the significantly higher median number of pathogen species observed in patients with severe immunosuppression illustrates a state of “microbiological chaos” resulting from the failure of immune surveillance. This metric of co-infection complexity serves as a quantifiable biomarker for the degree of underlying immune dysfunction. Conversely, in a state of severe immunosuppression, the collapse of immune surveillance and clearance mechanisms creates a favorable environment for the proliferation of multiple pathogens (Handley and Hand, 2022). This finding underscores that for patients with low CD4^+^ T cells count, anti-infective regimens must possess sufficient breadth to cover bacterial, fungal, and viral possibilities. Therapy targeting a single pathogen is often insufficient. The BALF-tNGS technique demonstrates its unique value in such patients; its ability to rapidly uncover the complex landscape of co-infections provides a critical evidence base for formulating comprehensive treatment strategies.

### 3.3 The correlation and implication of inflammatory response in relation to immune status

This study found that the immunocompetent patient group (Stratum 4) exhibited the highest levels of IL-6. We speculate that this is not coincidental but rather a reflection of their intact immune function. Previous mechanistic studies have demonstrated that in immunocompetent hosts, fully functional immune cells such as T cells and macrophages, upon recognizing pathogens, can be rapidly activated and produce large quantities of pro-inflammatory cytokines (including IL-6), thereby mounting a robust inflammatory response to clear the pathogen (O’Neill and Pearce, 2016). In contrast, immunocompromised patients have impaired immune cell function, which may lead to a delayed initiation or diminished intensity of the inflammatory response (Dilloo et al., 1995). Therefore, our observations reveal a potential profound link between the level of inflammation and immune competence, and a strong inflammatory response may indeed be a marker of intact immune function. This dissociation between immune competence and inflammatory response has important clinical implications. In immunocompromised patients, the absence of marked inflammation does not exclude serious infection, and conversely, elevated inflammatory markers in immunocompetent patients may represent appropriate immune mobilization rather than uncontrolled cytokine storm (Silva et al., 2023). This insight alerts us that when assessing infections in immunosuppressed patients, we cannot rely solely on the level of inflammatory markers to gauge disease severity. A low inflammatory response may indicate more profound immune paralysis, while a heightened inflammatory response could signify uncontrolled infection and inflammation. Future research should incorporate additional immunological parameters (e.g., monocyte and NK cell function) to enable more refined immune phenotyping.

### 3.4 Clinical implications and future perspectives

**Figure 6:**
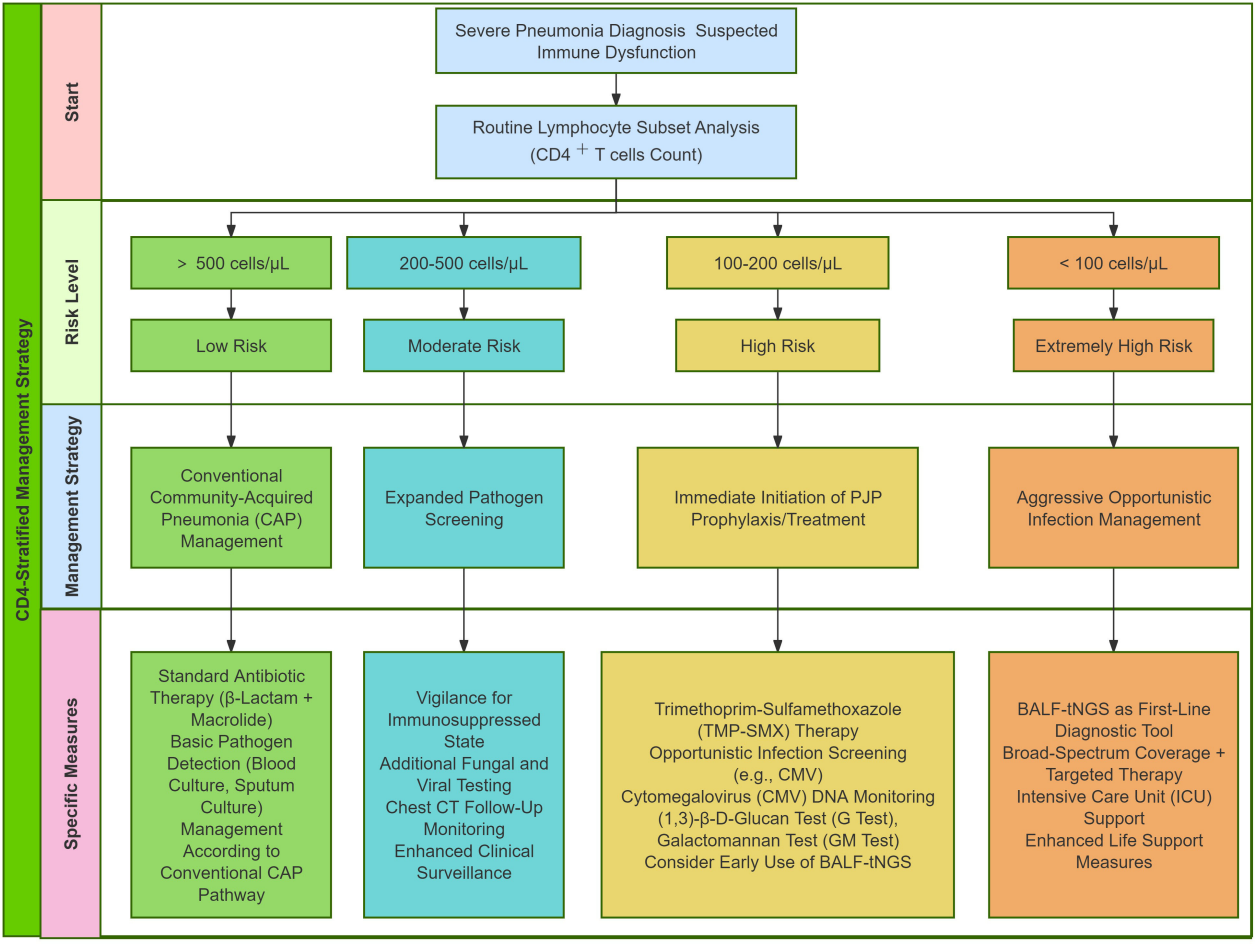
CD4^+^ T cells-stratified management pathway for severe pneumonia with suspected immune dysfunction.

Based on our findings, we propose a CD4^+^ T cells count-guided diagnostic and therapeutic pathway for severe pneumonia (**Figure 6**). We recommend that lymphocyte subset analysis, including CD4^+^ T cells count, be routinely incorporated into the initial evaluation of patients with severe pneumonia, especially when immune dysfunction is suspected. Clinical management should then be stratified according to the CD4^+^ T cells count as follows. In patients with CD4^+^ T cells count ≥ 500 cells/μL, management may adhere to standard guidelines for community-acquired pneumonia, as their immune competence is largely preserved. For those with a count between 200 and 500 cells/μL, clinicians should maintain a higher index of suspicion for underlying immunocompromise and consider expanding the spectrum of pathogen detection in diagnostic evaluations. In patients with CD4^+^ T cells count below 200 cells/μL—particularly under 100 cells/μL—imemptive prophylaxis against PJP pneumonia (e.g., trimethoprim-sulfamethoxazole) is strongly indicated, accompanied by active screening for cytomegalovirus and other opportunistic infections. In this high-risk group, BALF-tNGS may serve as a valuable complementary diagnostic tool. However, its results must be interpreted in conjunction with clinical presentation and ancillary microbiologic data to accurately distinguish true infection from colonization or latent viral shedding.

### 3.5 Study limitations and conclusion

This study has several limitations. First, as a single-center retrospective study, it is susceptible to selection bias, and the findings are inherently susceptible to selection bias and may lack generalizability to broader populations. To strengthen the validity of our conclusions, future multicenter prospective studies are warranted to validate the association between CD4^+^ T cells stratification and pathogen profiles across diverse clinical settings. Additionally, while BALF-tNGS excels at deep sequencing of specific targets, it has inherent drawbacks. The primary and most fundamental limitation is its finite amount of information. BALF-tNGS can only detect genes or regions that are pre-designed on its panel, making it incapable of discovering novel targets outside the panel, such as entirely new pathogens or unknown disease-causing genes. This renders it ineffective when confronted with unknown scenarios (Chen et al., 2024). Moreover, careful interpretation of BALF-tNGS results is crucial to avoid misdiagnosis. For negative results, clinicians should consider the possibility of pathogens not covered by the panel or below the detection threshold. For detected pathogens, especially those with known colonization potential (e.g., Candida, EBV), clinical correlation is essential. Results should be interpreted in conjunction with the host’s immune status, radiographic findings, and other laboratory tests to distinguish between true infection and colonization or latency. Secondly, the lack of uniform standards for panels, procedures, and databases across different laboratories makes it difficult to compare and standardize results directly. These factors collectively limit the broader application of BALF-tNGS (Society of Clinical Microbiology of China International E, Promotion Association for M, Healthcare, 2024).

In conclusion, this study confirms that the CD4^+^ T cells count is a key variable for the pathogen profile in severe pneumonia. A descending CD4^+^ T cells gradient is associated with a significantly increased risk of opportunistic pathogens and greater infection complexity. Integrating CD4^+^ T cells counting into the initial assessment framework for severe pneumonia could facilitate more precise and earlier targeted therapy, ultimately improving patient outcomes.

## Data Availability

The original contributions presented in the study are included in the article/supplementary material, further inquiries can be directed to the corresponding author/s.
